# Local neural-network-weighted models for occurrence and number of down wood in natural forest ecosystem

**DOI:** 10.1038/s41598-022-10312-x

**Published:** 2022-04-16

**Authors:** Yuman Sun, Weiwei Jia, Wancai Zhu, Xiaoyong Zhang, Subati Saidahemaiti, Tao Hu, Haotian Guo

**Affiliations:** 1 Key Laboratory of Sustainable Forest Ecosystem Management-Ministry of Education, School of Forestry, Northeast Forestry University, Harbin, 150040 China; 2 School of Forestry, Northeast Forestry University, Harbin, 150040 China; 3 Heilongjiang Forestry Institute, Harbin, 150081 China

**Keywords:** Ecology, Environmental sciences

## Abstract

The natural forest ecosystem has been affected by wind storms for years, which have caused several down wood (DW) and dramatically modified the fabric and size. Therefore, it is very important to explain the forest system by quantifying the spatial relationship between DW and environmental parameters. However, the spatial non-stationary characteristics caused by the terrain and stand environmental changes with distinct gradients may lead to an incomplete description of DW, the local neural-network-weighted models of geographically neural-network-weighted (GNNWR) models are introduced here. To verify the validity of models, our DW and environmental factors were applied to investigate of occurrence of DW and number of DW to establish the generalized linear (logistic and Poisson) models, geographically weighted regression (GWLR and GWPR) models and GNNWR (GNNWLR and GNNWPR) models. The results show that the GNNWR models show great advantages in the model-fitting performance, prediction performance, and the spatial Moran’s I of model residuals. In addition, GNNWR models can combine the geographic information system technology for accurately expressing the spatial distribution of DW relevant information to provide the key technology that can be used as the basis for human decision-making and management planning.

## Introduction

In the natural forest environment of northeast China, down wood (DW) is often caused by natural factors, which are generally divided into biological and abiotic factors^1^. Biological factors are caused by the age of trees, unbalanced growth, consumption of animals, diseases, etc^2^. The wind, fire, lightning, environmental stress (floods, droughts, and high temperatures), chemical pollution, and climate change are the main abiotic factors^3,4^. Liangshui National Nature Reserve is located in Xiaoxing’an mountains; the wind is considered the main reason for a mass of DW, which can cause the breakage or uprooting of living trees. The loss of living trees will have a serious impact on the economy^5–7^. In addition, the ecological significance of DW cannot be ignored^8,9^. DW plays key roles in resource cycling^10^, maintaining biodiversity^11–13^ and changing the micro-environment of stand^7,14,15^. Hence, it is distinctly indispensable to predict the spatial distribution of occurrence and number of DW in natural forests.

At present, many scholars have carried out a great deal of work on the DW^16^, among which, it shows more advantages of building theoretical models for the sake of researching spatial distribution of DW, geographical environment, stand characteristics, and other driving factors. Generalized linear (GL) models are widely applied in predicting the probability occurrence and mathematical statistics of events^17,18^. Among them, logistic regression^19,20^ is widely applied in predict the occurrence of DW (ODW), and Poisson regression^21^ is widely applied in predict the number of DW (NDW) caused by forest ecological environment attributes. However, GL models assume that the global space is stable, but it is difficult to find the data that satisfies this condition in forestry and the ecological environment. The space of Tobler says, “Distance is an important factor in whether or not things are related^22,23^.” This fact suggests that tree growth and stand development are highly likely to be influenced by spatial effects (spatial non-stationarity) of adjacent stands and that many ecological processes (such as DW) follow similar rules in space^24^. Therefore, British scholars Brunsdon and Fotheringham^25^ based on the spatial coefficient of variation, the geographically weighted regression (GWR) model is proposed^25^. This model method has been applied to various industries and has good results^26,27^. The fitting result of GWR model is related to the choice of bandwidth and weighting functions^28,29^.

GWR can well support the analysis of spatial non-stationarity in a dynamic environment, but complex geographic processes are difficult to model^30,31^. First, GWR cannot select the most accurate kernel function and determine the correct kernel function through the cross-validation method, and it is unable to estimate the spatial non-stationarity in a complex geographical environment accurately^32^. Second, the form of GWR kernel function needs to satisfy the conditions of a priori hypothesis. Third, the relative simplicity of the kernel function can only solve simple nonlinear problems but cannot solve complex nonlinear interactions in the geographical environment^33,34^. For this reason, Du^35^ proposed the use of the spatially weighted neural network (SWNN) to replace the kernel matric for the spatial coefficient of variation construction to perform and put forward a GNNWR model to solve complex non-stationary and nonlinear problems in coastal environments. Further, the instability of the forest ecosystem in the process of the ecological environment poses a similar issue. In this paper, we study SWNN combined with logistic regression and Poisson regression models, respectively, a geographically neural-network-weighted logistic regression (GNNWLR) model and a geographically neural-network-weighted Poisson regression (GNNWPR) model are introduced.

We tend to think about the Liangshui National Nature Reserve as the study area and established the relationship between a series of terrain and stand variables on the ODW (binary) and NDW (count). They include (1) logistic regression (GL, GWLR, and GNNWLR) models and Poisson regression (GL, GWPR, and GNNWPR) models to terrain and stand variables, respectively, in order to predict the ODW and NDW; (2) compare the performance of different models fitting, predicting, and residual Moran’s I; (3) the spatial distribution law of model coefficients in different geographical locations was intuitively displayed and analyzed by the GIS technology.

## Materials

### Study area

**Figure 1:**
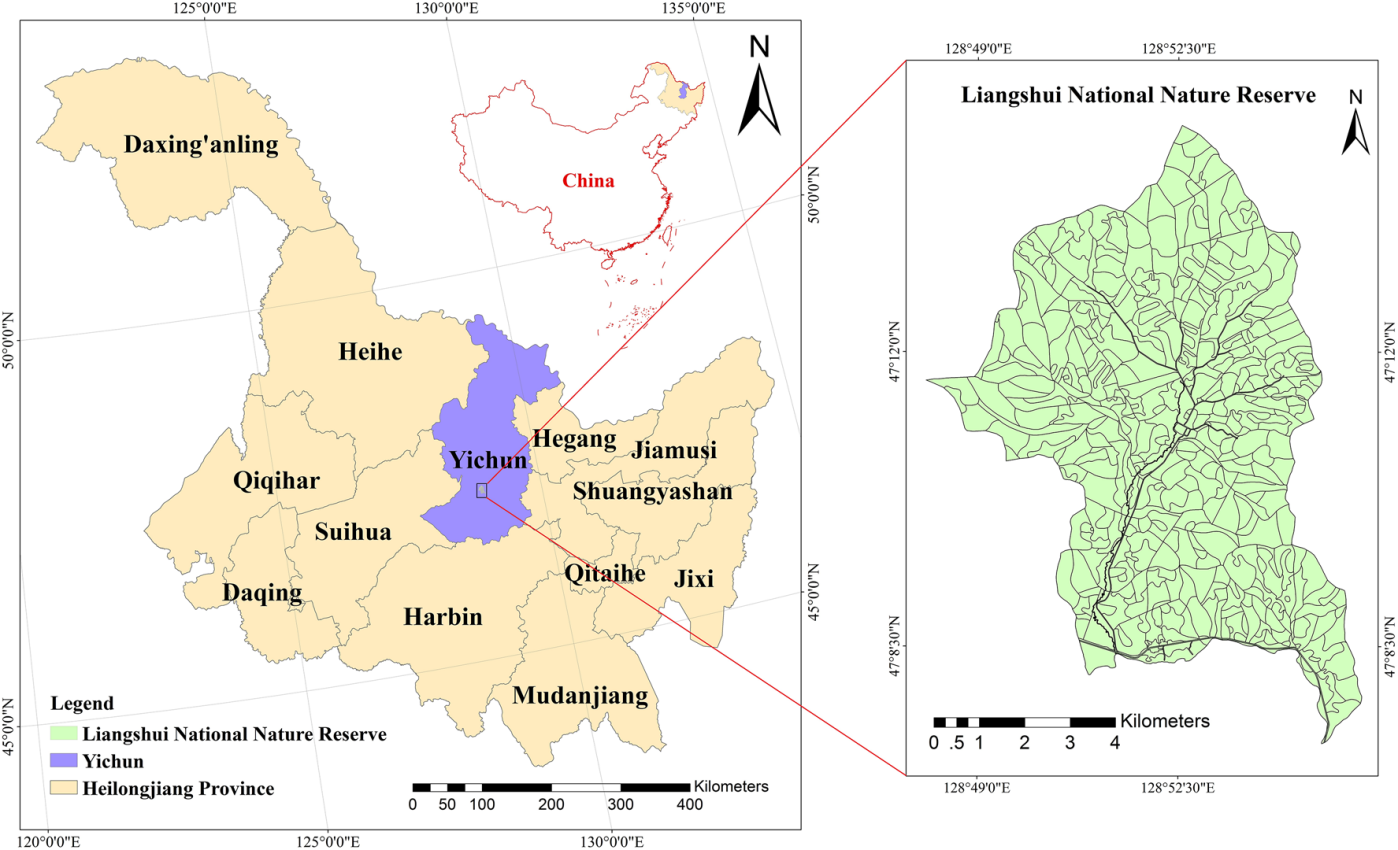
Study area (Liangshui National Nature Reserve). ArcGIS10.4 was used to draw the maps.

Liangshui National Nature Reserve is affiliated with the Northeast Forestry University, and the study area is located in Yichun City of northeast China (Fig. 1). It belongs to the southeast section of the Xiaoxing’an mountains range, the eastern slope of the Daridailing branch, with a gross area is 12,133 ha, the core area of 6394 ha is the study area. There are many primitive *Pinus koraiensis* preserved in China and secondary *birch* and broad-leaved forests covering different succession stages of mixed broad-leaved primitive *Pinus koraiensis* forests. Other major tree species includes *Spruce, Fir, Juglans mandshurica, Betula platyphylla*, etc.

The data comes from the 2019–2020 Forest Resources Planning and Design Survey. There are 32 compartments and 464 sub-compartments; 31 compartments and 443 sub-compartments belong to the arbor forest land. We have also carried out down deadwood volume (including down wood, standing deadwood, and others) surveys on the plots^36^. In order to further manage the area, according to the actual situation, we investigated the occurrence and number of valuable down wood through corner gauge points to sub-compartments in this research. Meanwhile, the tree species, number of living trees (NLT), forest stand mean height (H), and diameter at breast height (DBH) in sub-compartments were summarized by using an angle gauge. Moreover, terrain factors, such as DEM (m), slope (°), aspect and stand factors, mean age of living trees (years), canopy, vegetation coverage (%), etc., are also recorded here.

### Variable selection

This study aims to utilize terrain and stand variables to introduce independent and dependent variable models. The independent variables are ODW (binary) and the NDW (count). The final retention was determined by using stepwise regression (significance level $\alpha = 0.05$)^37,38^ and following five dependent variables: the number of living trees (NLT), canopy, forest stand mean height (H), slope, and forest stand mean DBH (DBH). Table 1 describes statistical variables related to statistical techniques.

**Table 1:** Description of the basic statistics of dependent variables and independent variables.

Dependent variables and independent variables	Num	Min	Mean	Std	Max
Number of down wood NDW (n/ha)	443	0	12.56	19.49	120
Occurrence of down wood ODW	443	0	0.54	0.50	1
Number of living trees NLT (n/ha)	443	191	968.24	483.23	3006
Canopy	443	0.40	0.66	0.10	0.90
Forest stand mean height H (m)	443	1.30	18.69	3.75	29.20
Slope (°)	443	2	10.75	4.66	25
Forest stand mean DBH DBH (cm)	443	3.00	25.12	11.19	48.00

**Figure 2:**
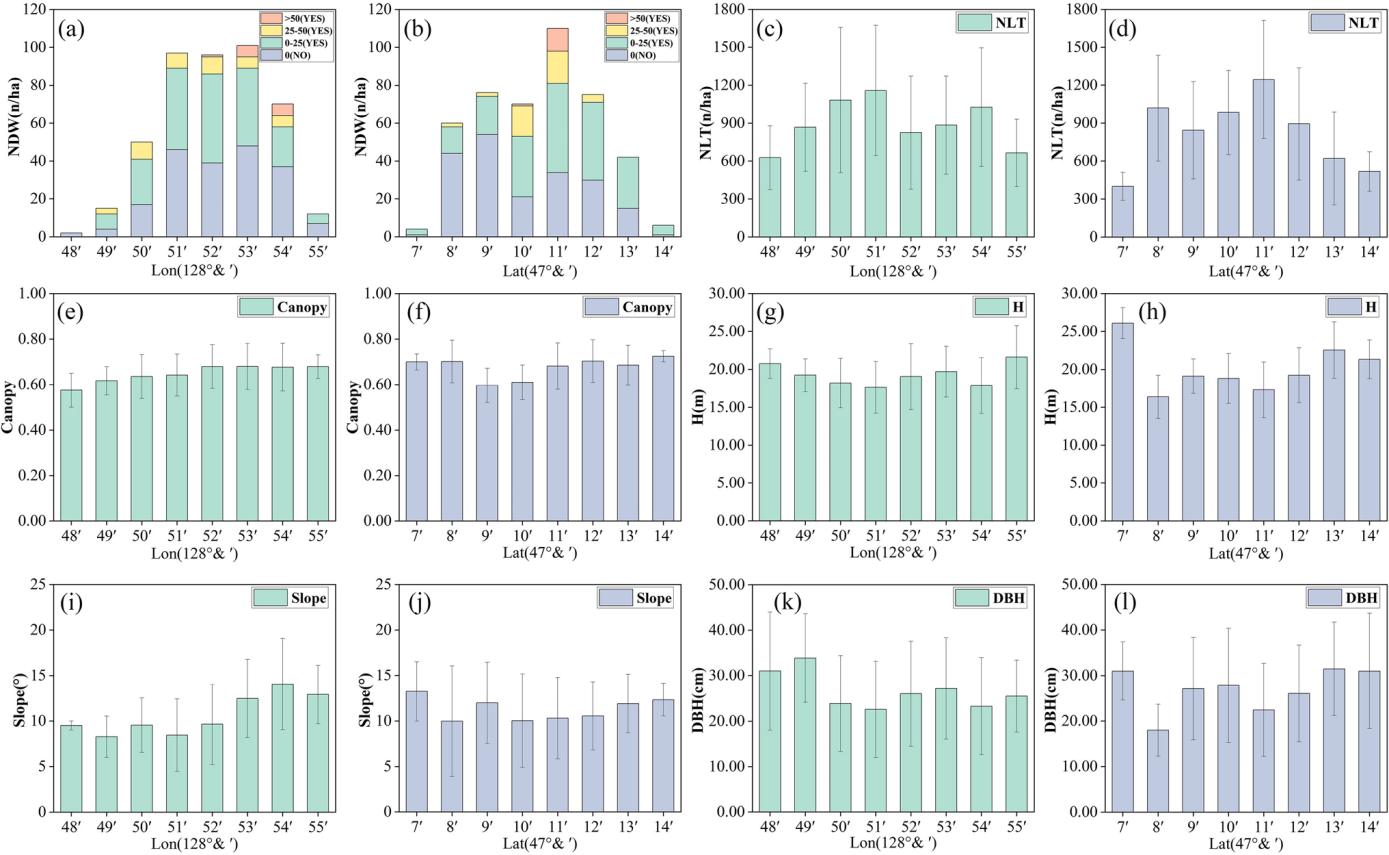
The trend of each variable along the longitude and latitude.

In order to study the non-stationarity in each direction, the variation of each variable along the longitude and latitude is calculated in Fig. 2. Considering the NDW, the incidence of the DW under different longitudes, latitudes, and the affect DW variables under any latitudes and longitudes, the following results are observed: the probability of more than half, the NDW is observed commonly in 0–25 n/ha, with an increase in the longitude the phenomenon of the DW appears for more than 50 n/ha, and DW more frequently in middle latitudes in the region, but cannot clear the trend of change. Moreover, it can be seen from the figure that the variation trends of the longitude and latitude of the other five independent variables are also inconsistent. Further, the longitude of the slope gradually increases and along the latitude of slope gradually decreases and then increases, its value is the lowest in the mid-latitude region. In conclusion, the variation of each variable significantly changes along the longitude and latitude, which further indicates that the stand environment shows high spatial non-stationarity.

## Methods

### GL models

In many practical cases, the response variable is essentially binary^39,40^. That is, there are two possibilities in the result, namely, which are assigned a value of 0 (does not happen) or 1 (does happen). Here, we set ODW (*y* = 1) as *P*, and no ODW (*y* = 0) as 1−*P*. Logistic regression between the occurrence probability of the DW and their respective variables was established as shown in Eq. (1)^20,41^:

1 $$Log{\text{it}}(P) = \ln (P/(1{ - }P)) = \beta_{0} + \sum\nolimits_{k = 1}^{5} {\beta_{k} x_{ik} }$$

where P represents the probability of the ODW and $\beta_{0} \sim \beta_{k}$ represents the regression coefficient of the model. k is the number of independent variables.

The Poisson model is usually attributed to count data. Here, the Poisson model can be utilized to predict the NDW, in line with the Poisson variable $Y$ and the Poisson probability density distribution function $f(Y,\mu ) = e^{ - \mu } \mu^{Y} /Y!$, where $\lambda$ is the mathematical expectation and variance of the random variable $Y$, namely, $E(Y) = \lambda$ and $V{\text{ar}}(Y) = \lambda$; a monotonous average link function of response variables as a linear model is obtained by inducing some changes as shown in Eq. (2)^21^:

2 $$Log(E(Y)) = L{\text{o}}g\mu = \beta_{0} + \sum\nolimits_{k = 1}^{5} {\beta_{k} } x_{ik}$$

It is assumed that the observed values are independent of each other, where $\mu$ represents the NDW and $\beta_{0} \sim \beta_{k}$ represents the regression coefficient of the model. k is the number of independent variables. All models estimate $\beta_{0} \sim \beta_{k}$ by the maximum likelihood method.

### GWR models

However, the above GL (logistic and Poisson) models are global in nature. The data collected in different geographical locations shows completely different results in the actual forestry survey due to the interference of different geographical environments and stand factors^42^. In the GL models analysis, it is often assumed that the estimated value of model coefficients are independent of the geographic location of the collected data, which leads to the estimated results tending to an average value. Thus, it can be inferred that all sample locations are based on unbiased estimates^43^. Therefore, the GL models show certain limitations in their application^35^. At present, GWR models are commonly used for improving the non-stationarity of space^44,45^.

Among them, we use the geographically weighted logistic regression (GWLR) model^46,47^ to forecast the ODW. Eq. (3) is expressed as follows:

3 $$Log{\text{it}}(P(u_{i} ,v_{i} )) = \ln (P(u_{i} ,v_{i} )/(1{ - }P(u_{i} ,v_{i} ))) = \beta_{0} (u_{i} ,v_{i} ) + \sum\nolimits_{k = 1}^{5} {\beta_{k} (u_{i} ,v_{i} )X_{ik} }$$

where $\beta_{0} (u_{i} ,v_{i} )\sim \beta_{k} (u_{i} ,v_{i} )$ represents the coefficient of GWLR at the position $i$.

The geographically weighted Poisson regression (GWPR) model^48^ to forecast the NDW. Eq. (4) is expressed as follows:

4 $$\begin{gathered} Log(E(Y(u_{i} ,v_{i} ))) = L{\text{o}}g\mu (u_{i} ,v_{i} ) = \beta_{0} (u_{i} ,v_{i} ) + \sum\nolimits_{k = 1}^{5} {\beta_{k} (u_{i} ,v_{i} )X_{ik} } \hfill \\ \hfill \\ \end{gathered}$$

where $\beta_{0} (u_{i} ,v_{i} )\sim \beta_{k} (u_{i} ,v_{i} )$ represents the coefficient of GWPR at the position $i$. All models estimate $\beta_{0} \sim \beta_{k}$ by the maximum likelihood method.

### GNNWR models

However, the kernel function of GWR models is relatively simple, and it is not easy to accurately model the complex stand geographical environment. For this reason, we propose a geographically neural-network-weighted regression (GNNWR) model, which is similar to the GWR models and uses the form of neural networks for defining the spatial non-stationary relationship^35^. Here, we integrate GWR into GL models, and the GNNWLR and GNNWPR models are shown in Eqs. (5) and (6):

5 $$\begin{gathered} Log{\text{it}}(P \times w(u_{i} ,v_{i} )) = \ln (P \times w(u_{i} ,v_{i} )/(1{ - }P \times w(u_{i} ,v_{i} ))) \hfill \\ \begin{array}{*{20}c} {\begin{array}{*{20}c} {\begin{array}{*{20}c} {} & {} \\ \end{array} } & {} \\ \end{array} } & {} & {} & {} \\ \end{array} = \beta_{0} \times w_{0} (u_{i} ,v_{i} ) + \sum\nolimits_{k = 1}^{5} {\beta_{k} \times w_{k} (u_{i} ,v_{i} )X_{ik} } \hfill \\ \end{gathered}$$

where $w_{0} (u_{i} ,v_{i} )\sim w_{k} (u_{i} ,v_{i} )$ are the weight estimated at $\beta_{0} \sim \beta_{k}$ by using the corresponding logistic regression by the maximum likelihood method.

6 $$\begin{gathered} Log(E(Y \times w(u_{i} ,v_{i} ))) = L{\text{o}}g(\mu \times w(u_{i} ,v_{i} )) \hfill \\ \begin{array}{*{20}c} {\begin{array}{*{20}c} {\begin{array}{*{20}c} {\begin{array}{*{20}c} {} & {} \\ \end{array} } & {} \\ \end{array} } & {} \\ \end{array} } & {} & {} & {} \\ \end{array} = \beta_{0} \times w_{0} (u_{i} ,v_{i} ) + \sum\nolimits_{k = 1}^{5} {\beta_{k} \times w_{k} (u_{i} ,v_{i} )X_{ik} } \hfill \\ \end{gathered}$$

where $w_{0} (u_{i} ,v_{i} )\sim w_{k} (u_{i} ,v_{i} )$ are the weight estimated at $\beta_{0} \sim \beta_{k}$ by using the corresponding Poisson regression by the maximum likelihood method.

The spatial weight $w(u_{i} ,v_{i} )$ is expressed as shown in Eq. (7):

7 $$w(u_{i} ,v_{i} ) = \left[ {\begin{array}{*{20}c} {w_{0} (u_{i} ,v_{i} )} & 0 & 0 & 0 \\ 0 & {w_{1} (u_{i} ,v_{i} )} & 0 & 0 \\ 0 & 0 & {...} & 0 \\ 0 & 0 & 0 & {w_{5} (u_{i} ,v_{i} )} \\ \end{array} } \right]$$

In the GWR models, the spatial weights include the Gaussian function, double square function, etc., but their structure is relatively simple, so it is difficult to capture the complex relationship between the spatial distance and the non-stationary weight. Here, the calculation of the SWNN can be deemed to a complex nonlinear problem between the spatial distance and the weight^33,34^. The SWNN is utilized to develop the non-stationary weight matrix, and kernel weight is determined as expressed in Eq. (8):

8 $$w(u_{i} ,v_{i} ) = SWNN\left( {\left[ {d_{i1}^{S} ,d_{i2}^{S} , \cdots ,d_{in}^{S} } \right]^{T} } \right)$$

where $\left[ {d_{i1}^{S} ,d_{i2}^{S} , \cdots ,d_{in}^{S} } \right]$ is the distance between point $i$ and other samples.

The neural network is designed using the Keras deep learning framework, also known as the deep neural network^49,50^. By setting the initial learning rate, the information between adjacent layers is considered fully connected (FC) and passed to the hidden layers^51–53^ and the dropout algorithm is required to be used in the iterative process of the training models^54^. Here, we also set the batch size as the sample number of each training, defined as LeakyReLU^55^ a nonlinear activation function for each network layer. The expressions for each layer are as expressed in Eq. (9).

9 $$y_{l} = \sigma (w_{l}^{t} x_{l} + b_{l} )$$

where $l$ is the number of all the layers in the training, $x_{l}$ are features of input layers, $w_{l}^{t}$ is the weight matrix, $b_{1}$ is the offset parameter vector, $y_{l}$ are features of output layers, and $\sigma$ is the activation function.

**Figure 3:**
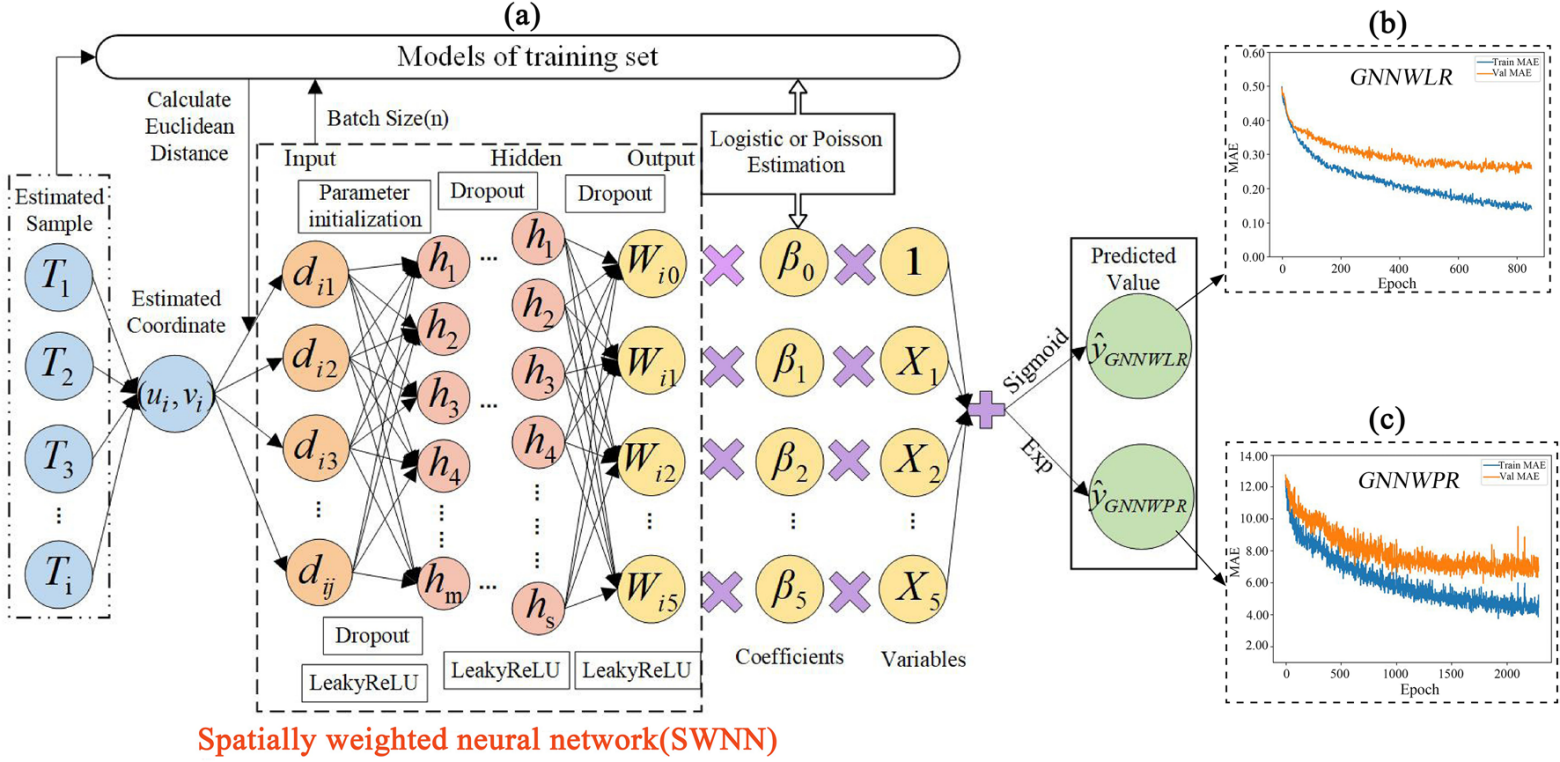
GNNWR (GNNWLR and GNNWPR) with the estimation of the design framework (**a**) MAE of epoch for GNNWLR (**b**) and GNNWPR (**c**).

When the training process encounters the triggering condition of early stop or the time of epoch reaches the set maximum, the training is stopped. Once the training process is completed, the prescient capacity of the model is estimated by utilizing a validation set. In this paper, Huber^56^ was used as the training loss function of the model, and the mean absolute error (MAE) of the validation set was used as the over-fitting evaluation index. By setting the maximum epoch, the trend of the MAE of the training set and the validation set was analyzed to find the optimal model parameters under the optimal number of iterations. To this end, its overall design framework of GNNWR (GNNWLR and GNNWPR) is shown in Fig. 3. Hyper-parameter settings of GNNWLR and GNNWPR models are shown in Table 2.

**Table 2:** Hyper-parameter settings of GNNWLR and GNNWPR.

Models	Input	Hidden1	Hidden2	Hidden3	Hidden4	Hidden5	Drop out	Learning Rate	Batch size	Max epoch	Stop epoch
GNNWLR	443	512	512	256	128	64	0.2	0.0005	10	20,000	850
GNNWPR	443	512	128	64	32	–	0.2	0.0008	20	20,000	2290

### Model assessment

The model's performance is assessed by utilizing the coefficient of determination (R^2^), which is utilized to assess the variability of the estimates, the standard deviation of the root-mean-square error (RMSE), and MAE estimates of the prediction error. The accuracy (acc) of 0 and 1 prediction under different logistic models was also analyzed. Moreover, the revised Akaike information criterion (AICc) is utilized to select the best bandwidth of the GWR models. The residual of the model is defined as the difference between the observed values and the predicted values, and the spatial autocorrelation of each model residuals was analyzed by using correlation graphs of global Moran’s I coefficients across a lag distance^36,57–59^.

## Results

### Model assessment

A total of 355 samples were randomly selected from 443 samples of the study area for model fitting and 88 samples for model validation. The standardized independent variables are NLT, canopy, H, slope, and DBH. The accuracy results are shown in Table 3. It can be seen that for both the training set and the validation set, the GNNWR (GNNWLR and GNNWPR) models are superior than GWR (GWLR and GWPR) models, and the GWR models are better than GL (Logistic and Poisson) models.

**Table 3:** Model accuracy verification statistics.

Models		Training set					Validation set				
		R^2^	RMSE	MAE	0 (Acc)	1 (Acc)	R^2^	RMSE	MAE	0 (Acc)	1 (Acc)
GL	Logistic	0.08	0.48	0.45	0.41	0.73	0.02	0.49	0.47	0.43	0.72
	Poisson	0.02	18.84	12.82	–	–	0.01	22.30	14.90	–	–
GWR	GWLR	0.49	0.35	0.29	0.73	0.92	0.26	0.43	0.35	0.67	0.72
	GWPR	0.62	11.71	7.03	–	–	0.29	17.73	9.55	–	–
GNNWR	GNNWLR	0.76	0.24	0.12	0.94	0.98	0.30	0.42	0.25	0.83	0.74
	GNNWPR	0.90	6.17	2.93	–	–	0.54	14.36	7.02	–	–

**Figure 4:**
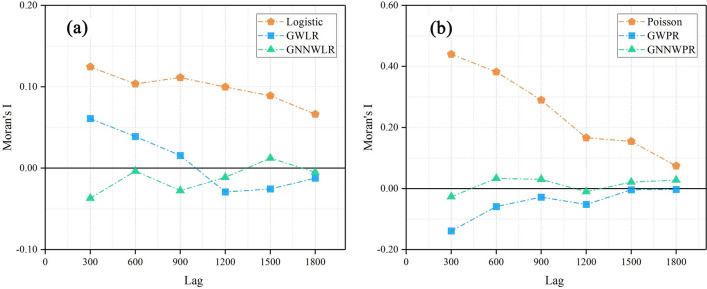
The spatial correlation between the residuals of ODW (**a**) and NDW (**b**) models.

In order to verify that the spatial effects of GNNWR models include spatial non-stationarity, comparing spatial effect processes of different models can reduce the ability to misleading significance testing and prediction models. Moran’s I and *Z*-value are calculated in Table 4. In order to better compare the spatial relationship of residuals of different models, spatial correlation graphs of residuals of different models are drawn with an interval of 300 m. Their average distance is about 300 m. Its neighbor pairs are 302, 1318, 2150, 2631, 3293 and 3751, respectively (Fig. 4).

**Table 4:** Moran’s I and Z-value for predicting ODW and NDW model residuals.

Models	GL		GWR		GNNWR	
	Logistic	Poisson	GWLR	GWPR	GNNWLR	GNNWPR
Moran’s I	0.12	0.44	0.06	− 0.14	− 0.05	− 0.03
Z-value	2.21	7.71	1.10	− 2.39	− 0.09	− 0.43

From Table 4, it can be seen that GL and GWPR models (*Z*-value > *Z* $\alpha /2$ = 1.96) indicate that the independent assumption of model residuals is contrary to these two types of models, and these results indicate a similar clustering pattern. In general, the ability to eliminate the autocorrelation of spatial residuals can be gleaned from the absolute value of *Z*-value, and it follows the order: GNNWR models > GWR models > GL models. It indicates that GNNWR models can effectively eliminate spatial non-stationarity. Moran’s I of GNNWR models are also relatively stable at any step size and close to 0, which indicates that GNNWR models demonstrate a good ability to maintain spatial stability.

### Model parameter analysis

According to the geographical location, the two types of models, GWR and GNNWR models, belong to local models and produce different geographical model coefficients. The descriptive statistics of the GWR and GNNWR model coefficients are summarized in Table 5. The distribution of GWR and GNNWR coefficients can be used to the non-stationary relationship between dependent and independent variables of stand environmental parameters^33,34^. The GWR and GNNWR models' coefficients show positive and negative fluctuations, indicating that the influence of the stand environment on DW may show opposite effects in different locations. Furthermore, the variation of the GNNWR coefficient is more dramatic than that of the response GWR coefficients, which may be the reason why the fitting and prediction performance of GNNWR has dropped significantly.

**Table 5:** Basic statistic parameters of GWR and GNNWR models.

Models	Coefficient	Mean	Min	Q1	Median	Q3	Max
GWLR	Intercept	0.08	− 2.77	− 0.13	0.31	0.91	2.15
	NLT	− 0.51	− 2.65	− 0.96	− 0.51	− 0.09	2.22
	Canopy	0.61	− 0.79	0.32	0.72	0.92	1.90
	H	− 0.41	− 2.96	− 1.35	− 0.57	0.33	3.67
	Slope	0.14	− 1.15	− 0.04	0.11	0.39	1.14
	DBH	0.44	− 0.52	0.11	0.45	0.73	1.51
GWPR	Intercept	1.65	− 13.04	1.40	2.28	2.78	3.54
	NLT	− 0.27	− 4.01	− 0.69	− 0.31	0.19	2.86
	Canopy	0.20	− 1.45	− 0.25	0.02	0.46	6.17
	H	− 0.01	− 1.90	− 0.82	− 0.35	0.14	14.90
	Slope	− 0.03	− 4. 41	− 0.20	0.04	0.19	1.72
	DBH	0.29	− 0.71	− 0.05	0.16	0.59	3.14
GNNWLR	Intercept	− 3.82	− 32.94	− 5.46	0.03	1. 87	11.55
	NLT	0.47	− 26.21	− 0.50	0.21	1. 55	9.08
	Canopy	− 0.54	− 25.55	− 2.00	− 0.11	1. 31	16.75
	H	1. 63	− 53.05	− 1.42	0.63	3. 38	44.85
	Slope	0.22	− 4.95	− 0.35	0.03	0.58	7.82
	DBH	− 0.52	− 25.87	− 2.45	− 0.47	0.77	22.91
GNNWPR	Intercept	0.47	− 6.90	− 1.45	1. 60	2. 82	3.95
	NLT	− 0.56	− 6.42	− 0.90	− 0.15	0.16	0.95
	Canopy	0.65	− 0.69	− 0.19	0.02	1.32	5.83
	H	− 0.52	− 5.36	− 0.90	− 0.24	0.25	1.39
	Slope	− 0.06	− 1.31	− 0.24	− 0.01	0.13	1.58
	DBH	0.96	− 1.59	0.02	0.41	1.43	5.55

### Visualized analysis of model parameters

**Figure 5:**
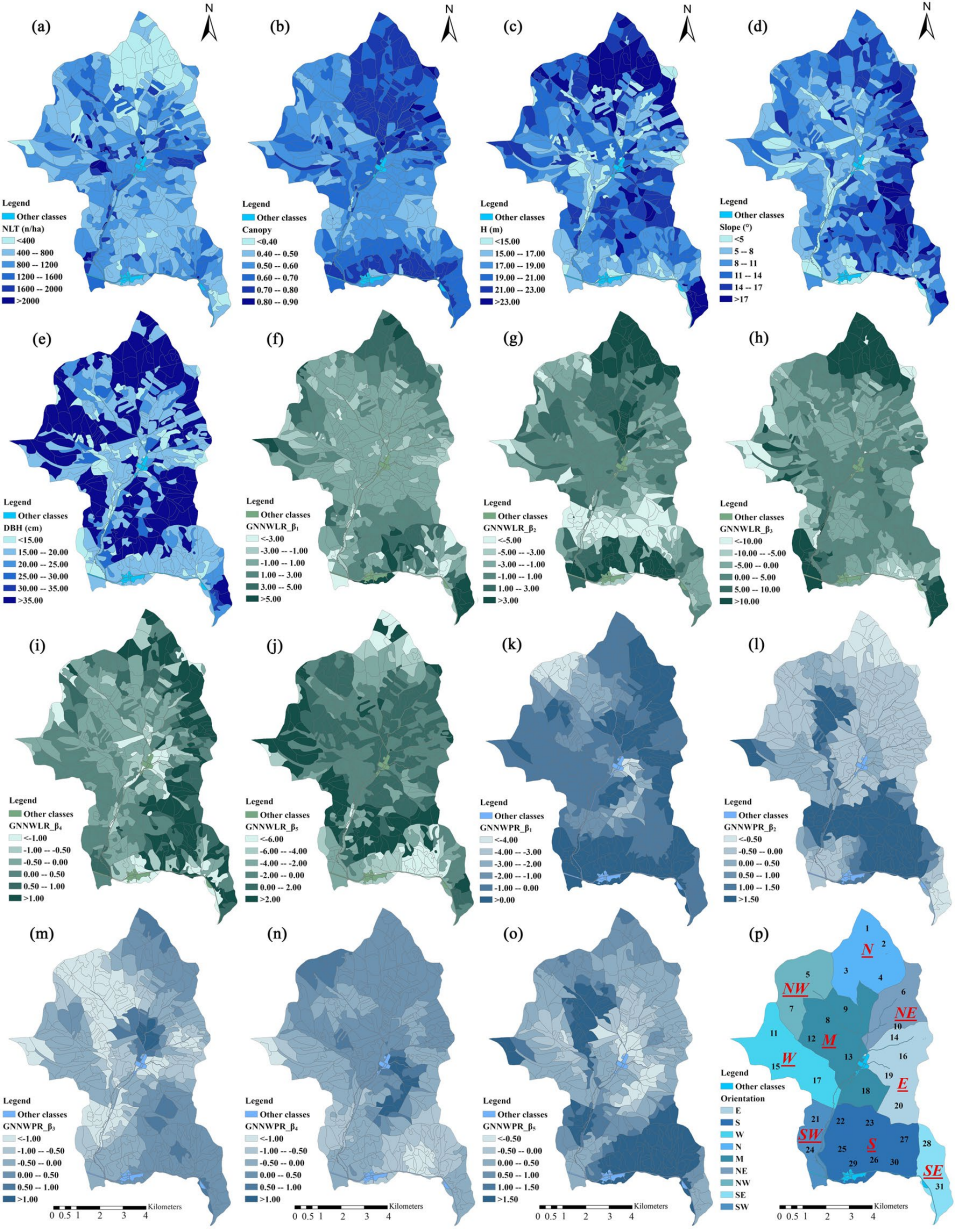
Spatial distribution of the 5 predictive variables NLT, canopy, H, slope, and DBH (**a**–**e**). Coefficient estimation of 5 predictive variables in GNNWLR (**f**–**j**) and GNNWPR (**k**–**o**) models. (**p**) is the divide the study area into 9 orientations. ArcGIS10.4 was used to draw the maps.

The visual distribution analysis of five predictive variables (NLT, canopy, H, slope, and DBH) is shown in Fig. 5a–e and according to the compartments. For further study the spatial correlation between ODW and NDW, the distribution diagrams of the model coefficients of the five predictor variables of the GNNWLR (Fig. 5f–j) and GNNWPR (Fig. 5k–o) models are presented. For the convenience of analysis, we divided the study area into nine orientations ((Fig. 5p). In terms of the NLT (Fig. 5a), there are fewer NLT in the N and S areas and more in the M area. Canopy (> 0.7) (Fig. 5b) is mainly distributed in the S and N areas; H (Fig. 5c) is higher in the N and SE. There are many rivers and roads in M area of Liangshui Nature Reserve. The slope in this area is slow (< 11°) (Fig. 5d). The DBH (Fig. 5e) of big living trees is > 35 cm and mainly clustered in N, W, and E areas. Therefore, it can be seen that in the N and SE areas, there are trees with less NLT and big DBH. The M area has medium-sized trees with a gentle slope. In the S area, there are more small trees with more NLT. Their slope is steep in the N, NE, E, and SE areas. The coefficients vary significantly in space and show several directional patterns, and the two symbols and sizes are often heterogeneous, which also explains the significant spatial non-stationarity of the DW space.

## Discussion

As the study area of Liangshui National Nature Reserve, there is a large DW affected by the wind storms, especially most of the trees are old primitive mixed broadleaf-conifer forest. The trees that have passed the mature stage become more and more vulnerable in the face of wind as the H taller, and the canopy grows larger, the physiological aging and the ability to resist diseases and insect pests decrease, and most of them end their life cycle in the state of breakage or uprooting^60,61^ and such disaster changed its native forest stand structure^62^, which is associated with the hardwood species changing into the broad-leaved mixed forest and shows the positive pioneer trees, such as *oak*, *birch*, and *aspen*^63^. This indicates the formation of the secondary forest. Not only that, but it also leads to the loss of a lot of valuable timber in this area^64^. Therefore, it shows the economic value to the region (not rotten wood), and buck and forest investigate the environmental factors by using the data of the ODW (binary)^19,20^ and the NDW (count)^21^ to establish the GL, GWR, and GNNWR models, respectively. To better understand spatial non-stationarity caused by DW, the terrain with obvious gradient change and stand environment are used in the study area.

**Figure 6:**
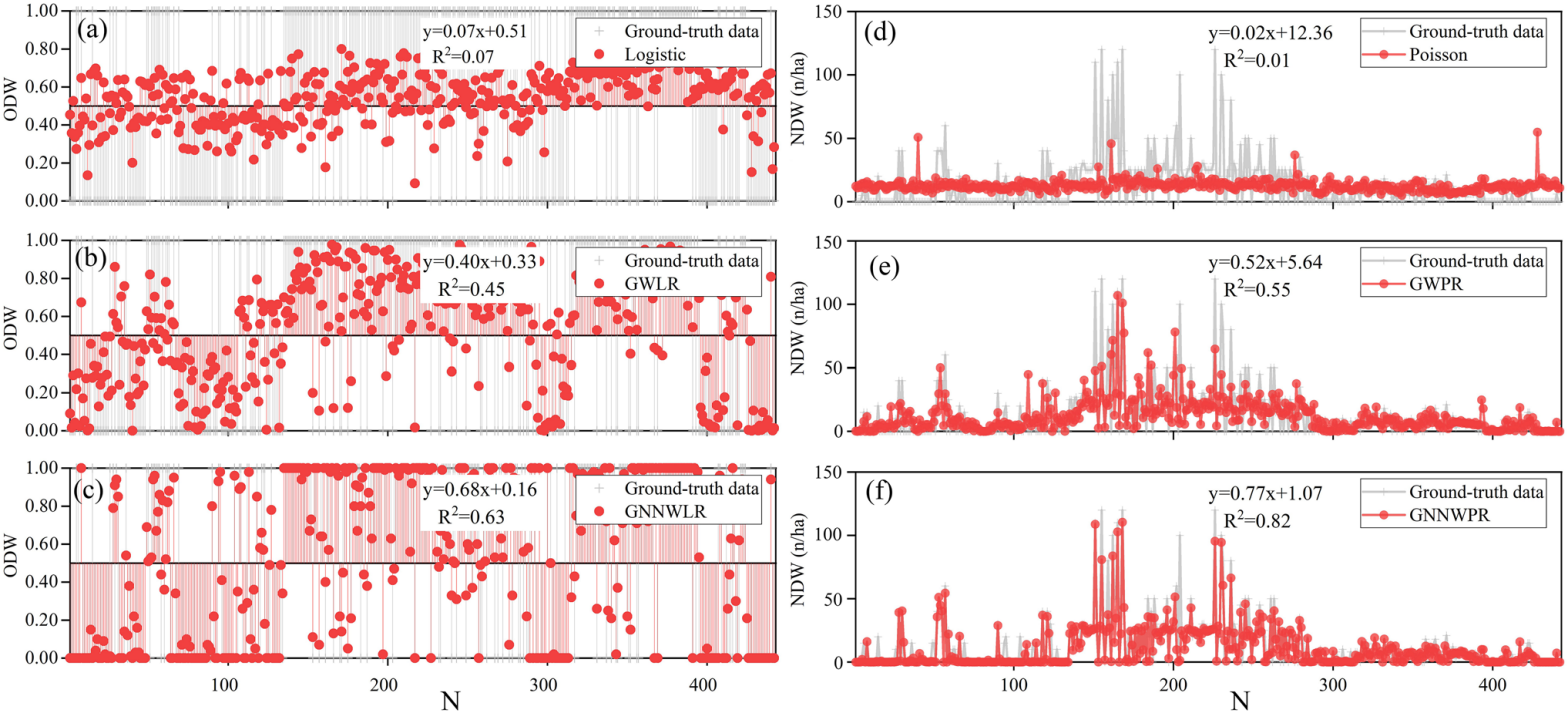
Correlation logistic and Poisson (GL models (**a**, **d**), GWR models (**b**, **e**), and GNNWR models (**c**, **f**)) and ground-truth data of comparative analysis.

Here, we compare the three methods and find that the non-stationary matrix constructed by the SWNN can well solve the complex nonlinear problem between the weight space distance and the weight space and thus can better predict the spatial change of the DW^33,34^. This method also has good results to solve complex non-stationary and nonlinear problems in coastal environments^33–35^. It is found that the GNNWR model shows absolute advantages in both the training set and the validation set ^35^. At the same time, we utilized Moran’s I and Z-value to check the non-stationarity of model space and observed that GL and GWR models would have independent assumptions and inefficient model coefficient estimates. In addition, according to the spatial residual correlation graph drawn at every 300 m, the Moran’s I of GNNWR models at any step size is relatively stable and close to 0, which indicates that GNNWR models show a very good ability to maintain spatial stability^65^. At this moment, drop lines (logistic regression) and point plot (Poisson regression) with the ground survey data, respectively, and draw its 1:1 linear fitting diagram. The results of GNNWR models are closest to the ground-truth data (Fig. 6).

The distribution of ODW and NDW are easily affected by the climate, stand, terrain, and tree factors^61^. For the investigation, variables in the Liangshui Nature Reserve, four stand variables (NLT, canopy, H, and DBH), and one terrain variable (slope)^66^ were selected by stepwise regression. NLT represents the density of the stand, and the canopy represents the size of the canopy structure. The DBH and H represent the tree size^67–69^; the slope represents the geographical characteristics of stands^66^.

**Figure 7:**
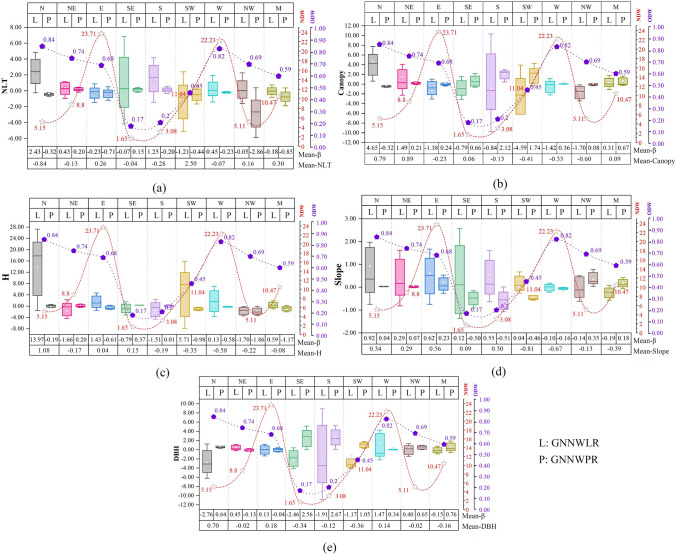
The statistical analysis of 5 normalized variables and coefficients of ODW and NDW in 9 orientations under GNNWR models.

In GNNWR models, the symbols and sizes of the model coefficients are often heterogeneous; that is, different stand environments show different or even opposite effects on the distribution of the DW^33,34^. Here, we analyze the 5 variables of GNNWLR and GNNWPR models and the 9 orientations of model coefficients(ODW and NDW), and we can judge whether they are positively or negatively correlated with the corresponding dependent variables from the positive and negative mean values of variable model coefficients. However, we can see from the range of the box that these are not the only relations (Fig. 7), which will vary according to different regions, which also shows the existence of non-stationarity in the spatial model^35^. In the E and W orientations, the ODW is high and the NDW is large^64^. In the N, NE and NW orientations, the ODW is high, but NDW not large. In the region of N and NE, NLT is less, but the trees size and the tree canopy are large and the slope is steep. However, in the NW area, trees are of medium size and more NLT. However, SE, S and SW orientations are not easy to have DW, and the NDW is small. The tree size is small, the canopy is big, NLT is medium, the slope is gentle. In the M orientation, there are certain ODW and NDW^63^.

Finally, the predicted ODW and NDW on the visualization distribution maps of GNNWR closest to the ground-truth results, combined with statistical graphics and GIS mapping ability^70^, can provide inverted wooden key visual information, and forest managers can distinguish areas that are affected by disaster in order to help the administrator to provide timber and forest terrain variables and the relationship between the detailed information. Therefore, GNNWLR models are used to evaluate the ODW in a given area or small class, and GNNWPR models are used to predict the NDW. In order to prevent wind damage in the future, stand density, structure, and species composition can be changed in local areas^63^, which can assist in decision making and management planning for rational afforestation and management activities in the natural forest ecosystem.

## Conclusion

Logistic regression (GL, GWLR, and GNNWLR) models and Poisson regression (GL, GWPR, and GNNWPR) models were used to model terrain variables and stand variables, respectively, for predicting the ODW and NDW. The analysis indicated that GNNWR models offer greater advantages than GWR and GL models in model-fitting and validation performance and also produced ideal residuals to validate spatial correlation. In addition, the GIS technology provides much useful information in the study area about different ODW and NDW caused by spatial non-stationarity to stand and terrain factors. For a given area, the spatial distribution information of the DW can be clearly identified, which can be considered a good approach to assess the damage caused by natural disasters, provide key information for forest resources, assist in decision making and management schemes, and avoid and reduce the disturbance and loss caused by natural disasters.

## Data Availability

If the manuscript is accepted, some data can be published.

## References

[1] Franklin JF, Shugart HH, Harmon ME (1987). Tree death as an ecological process. Bioscience.

[2] Harmon ME, MacFadyen A, Ford ED (1986). Ecology of coarse woody debris in temperate ecosystems. Advances in Ecological Research.

[3] Harmon ME, Bell DM (2020). Mortality in forested ecosystems: suggested conceptual advances. Forests.

[4] van Mantgem PJ (2009). Widespread increase of tree mortality rates in the Western United States. Science.

[5] Kinnucan HW (2016). Timber price dynamics after a natural disaster: Hurricane Hugo revisited. J. For. Econ..

[6] Marsinko AP, Straka TJ, Haight RG (1997). The effect of a large-scale natural disaster on regional timber supply. J. World For. Resour. Manag..

[7] Lugo AE (2008). Visible and invisible effects of hurricanes on forest ecosystems: an international review. Austral Ecol..

[8] Shifley SR, Brookshire BL, Larsen DR, Herbeck LA (1997). Snags and down wood in missouri old-growth and mature second-growth forests. North. J. Appl. For..

[9] Bobiec A (2002). Living stands and dead wood in the Białowieża forest: suggestions for restoration management. For. Ecol. Manag..

[10] Spetich MA, Shifley SR, Parker GR (1999). Regional distribution and dynamics of coarse woody debris in midwestern old-growth forests. For. Sci..

[11] Rimle A, Heiri C, Bugmann H (2017). Deadwood in Norway spruce dominated mountain forest reserves is characterized by large dimensions and advanced decomposition stages. For. Ecol. Manag..

[12] Ruokolainen A, Shorohova E, Penttilä R, Kotkova V, Kushnevskaya H (2018). A continuum of dead wood with various habitat elements maintains the diversity of wood-inhabiting fungi in an old-growth boreal forest. Eur. J. For. Res..

[13] Ranius T, Kindvall O (2004). Modelling the amount of coarse woody debris produced by the new biodiversity-oriented silvicultural practices in Sweden. Biol. Conserv..

[14] Bouget C, Duelli P (2004). The effects of windthrow on forest insect communities: a literature review. Biol. Conserv..

[15] Svensson M (2016). The relative importance of stand and dead wood types for wood-dependent lichens in managed boreal forests. Fungal Ecol..

[16] Bahuguna D, Mitchell SJ, Nishio GR (2012). Post-harvest windthrow and recruitment of large woody debris in riparian buffers on Vancouver Island. Eur. J. For. Res..

[17] Fortin M, DeBlois J (2007). Modeling tree recruitment with zero-inflated models: the example of hardwood stands in southern Quebec Canada. For. Sci..

[18] Herrero C, Pando V, Bravo F (2010). Modelling coarse woody debris in Pinus spp. Plantations. A case study in Northern Spain. Ann. For. Sci..

[19] Arekhi S (2011). Modeling spatial pattern of deforestation using GIS and logistic regression: a case study of northern Ilam forests, Ilam province Iran. Afr. J. Biotechnol..

[20] Kumar R, Nandy S, Agarwal R, Kushwaha SPS (2014). Forest cover dynamics analysis and prediction modeling using logistic regression model. Ecol. Indic..

[21] Podur JJ, Martell DL, Stanford D (2010). A compound poisson model for the annual area burned by forest fires in the province of Ontario. Environmetrics.

[22] Tobler WR (1970). A computer movie simulating urban growth in the Detroit Region. Econ. Geogr..

[23] Griffith D, Chun Y, Fischer MM, Nijkamp P (2014). Spatial autocorrelation and spatial filtering. Handbook of regional science 1477–1507.

[24] Li T, Meng Q (2019). Forest dynamics in relation to meteorology and soil in the Gulf Coast of Mexico. Sci. Total Environ..

[25] Brunsdon C, Fotheringham AS, Charlton ME (1996). Geographically weighted regression: a method for exploring spatial nonstationarity. Geogr. Anal..

[26] Fotheringham AS, Charlton ME, Brunsdon C (1998). Geographically weighted regression: a natural evolution of the expansion method for spatial data analysis. Environ. Plan. A.

[27] Yang C, Fu M, Feng D, Sun Y, Zhai G (2021). Spatiotemporal changes in vegetation cover and its influencing factors in the loess Plateau of China based on the geographically weighted regression model. Forests.

[28] Monjarás-Vega N (2020). Predicting forest fire kernel density at multiple scales with geographically weighted regression in Mexico. Sci. Total Environ..

[29] Peng X, Wu H, Ma L (2020). A study on geographically weighted spatial autoregression models with spatial autoregressive disturbances. Commun. Stat. Theor. Methods.

[30] Harris P, Brunsdon C (2010). Exploring spatial variation and spatial relationships in a freshwater acidification critical load data set for Great Britain using geographically weighted summary statistics. Comput. Geosci..

[31] Li J, Jin M, Li H (2019). Exploring spatial influence of remotely sensed PM2.5 concentration using a developed deep convolutional neural network model. Int. J. Environ. Res. Public Health.

[32] Peng C, Wang M, Chen W (2016). Spatial analysis of PAHs in soils along an urban-suburban-rural gradient: scale effect, distribution patterns, diffusion and influencing factors. Sci. Rep..

[33] Wu S (2021). Geographically and temporally neural network weighted regression for modeling spatiotemporal non-stationary relationships. Int. J. Geogr. Inf. Sci..

[34] Wu S (2020). Modeling spatially anisotropic nonstationary processes in coastal environments based on a directional geographically neural network weighted regression. Sci. Total Environ..

[35] Du Z, Wang Z, Wu S, Zhang F, Liu R (2020). Geographically neural network weighted regression for the accurate estimation of spatial non-stationarity. Int. J. Geogr. Inf. Sci..

[36] Sun Y, Ao Z, Jia W, Chen Y, Xu K (2021). A geographically weighted deep neural network model for research on the spatial distribution of the down dead wood volume in liangshui national nature reserve (China). IForest.

[37] Wilkinson L (1979). Tests of significance in stepwise regression. Psychol. Bull..

[38] Henderson DA, Denison DR (1989). Stepwise regression in social and psychological research. Psychol. Rep..

[39] Carl G, Kühn I (2007). Analyzing spatial autocorrelation in species distributions using Gaussian and logit models. Ecol. Model..

[40] Wu W, Zhang L (2013). Comparison of spatial and non-spatial logistic regression models for modeling the occurrence of cloud cover in north-eastern Puerto Rico. Appl. Geogr..

[41] Ozdemir A (2011). Using a binary logistic regression method and GIS for evaluating and mapping the groundwater spring potential in the Sultan Mountains (Aksehir, Turkey). J. Hydrol..

[42] Pineda Jaimes NB, Bosque Sendra J, Gómez Delgado M, Franco Plata R (2010). Exploring the driving forces behind deforestation in the state of Mexico (Mexico) using geographically weighted regression. Appl. Geogr..

[43] Tutmez B, Kaymak U, Erhan Tercan A, Lloyd CD (2012). Evaluating geo-environmental variables using a clustering based areal model. Comput. Geosci..

[44] Li X, Wu P, Guo F-T, Hu X (2021). A geographically weighted regression approach to detect divergent changes in the vegetation activity along the elevation gradients over the last 20 years. For. Ecol. Manag..

[45] Que X, Ma C, Ma X, Chen Q (2021). Parallel computing for fast spatiotemporal weighted regression. Comput. Geosci..

[46] Wu L (2016). Spatial analysis of severe fever with thrombocytopenia syndrome virus in China using a geographically weighted logistic regression model. Int. J. Environ. Res. Public Health.

[47] Liu Y (2020). Geographical variations in maternal lifestyles during pregnancy associated with congenital heart defects among live births in Shaanxi province Northwestern China. Sci. Rep..

[48] Saefuddin A, Saepudin D, Kusumaningrum D (2013). Geographically weighted poisson regression (GWPR) for analyzing the malnutrition data in java-Indonesia.

[49] Lecun Y, Bengio Y, Hinton G (2015). Deep learning. Nature.

[50] Ketkar N, Ketkar N (2017). Introduction to Keras. Deep learning with python: a hands-on introduction.

[51] Tsomokos DI, Ashhab S, Nori F (2008). Fully connected network of superconducting qubits in a cavity. New J. Phys..

[52] Hu T (2021). Study on the estimation of forest volume based on multi-source data. Sensors.

[53] Chen L, Ren C, Zhang B, Wang Z, Xi Y (2018). Estimation of forest above-ground biomass by geographically weighted regression and machine learning with sentinel imagery. Forests.

[54] Srivastava N, Hinton G, Krizhevsky A, Sutskever I, Salakhutdinov R (2014). Dropout: a simple way to prevent neural networks from overfitting. J. Mach. Learn. Res..

[55] Mastromichalakis, S. ALReLU: A different approach on Leaky ReLU activation function to improve neural networks performance. arXiv:2012.07564 [Cs] arXiv:2012.07564 (2021).

[56] Chen C, Li Y, Yan C, Dai H, Liu G (2015). A robust algorithm of multiquadric method based on an improved huber loss function for interpolating remote-sensing-derived elevation data sets. Remote Sens..

[57] de Jong P, Sprenger C, Veen F (1984). On extreme values of Moran’s I and Geary’s c ( spatial autocorrelation). Geogr. Anal..

[58] Fu WJ, Jiang PK, Zhou GM, Zhao KL (2014). Using Moran’s i and GIS to study the spatial pattern of forest litter carbon density in a subtropical region of southeastern China. Biogeosciences.

[59] Parizi E, Hosseini SM, Ataie-Ashtiani B, Simmons CT (2020). Normalized difference vegetation index as the dominant predicting factor of groundwater recharge in phreatic aquifers: case studies across Iran. Sci. Rep..

[60] Moore JR (2000). Differences in maximum resistive bending moments of Pinus radiata trees grown on a range of soil types. For. Ecol. Manag..

[61] Lanquaye-Opoku N, Mitchell SJ (2005). Portability of stand-level empirical windthrow risk models. For. Ecol. Manag..

[62] Li X (2018). Response of species and stand types to snow/wind damage in a temperate secondary forest Northeast China. J. For. Res..

[63] Zhen Z (2013). Geographically local modeling of occurrence, count, and volume of downwood in Northeast China. Appl. Geogr..

[64] Vozmishcheva A (2019). Strong disturbance impact of tropical cyclone Lionrock (2016) on Korean pine-broadleaved forest in the Middle Sikhote-Alin Mountain range Russian Far East. Forests.

[65] Bivand R, Müller WG, Reder M (2009). Power calculations for global and local Moran’s I. Comput. Stat. Data Anal..

[66] Yuan J (2017). Dynamics of coarse woody debris characteristics in the Qinling mountain forests in China. Forests.

[67] Næsset E (1997). Estimating timber volume of forest stands using airborne laser scanner data. Remote Sens. Environ..

[68] Næsset E (2002). Determination of mean tree height of forest stands by digital photogrammetry. Scand. J. For. Res..

[69] Rich RL, Frelich LE, Reich PB (2007). Wind-throw mortality in the southern boreal forest: effects of species, diameter and stand age. J. Ecol..

[70] Odhiambo BO, Kenduiywo BK, Were K (2020). Spatial prediction and mapping of soil pH across a tropical afro-montane landscape. Appl. Geogr..

